# Trait Anxiety Impacts the Perceived Gaze Direction of Fearful But Not Angry Faces

**DOI:** 10.3389/fpsyg.2017.01186

**Published:** 2017-07-14

**Authors:** Zhonghua Hu, Maria Gendron, Qiang Liu, Guang Zhao, Hong Li

**Affiliations:** ^1^Research Center of Brain and Cognitive Neuroscience, Liaoning Normal University Dalian, China; ^2^Department of Psychology, Northeastern University, Boston MA, United States; ^3^College of Psychology and Sociology, Shenzhen University Shenzhen, China

**Keywords:** trait anxiety, the cone of direct gaze (CoDG), facial expression, angry, fearful

## Abstract

Facial expression and gaze direction play an important role in social communication. Previous research has demonstrated the perception of anger is enhanced by direct gaze, whereas, it is unclear whether perception of fear is enhanced by averted gaze. In addition, previous research has shown the anxiety affects the processing of facial expression and gaze direction, but hasn’t measured or controlled for depression. As a result, firm conclusions cannot be made regarding the impact of individual differences in anxiety and depression on perceptions of face expressions and gaze direction. The current study attempted to reexamine the effect of the anxiety level on the processing of facial expressions and gaze direction by matching participants on depression scores. A reliable psychophysical index of the range of eye gaze angles judged as being directed at oneself [the cone of direct gaze (CoDG)] was used as the dependent variable in this study. Participants were stratified into high/low trait anxiety groups and asked to judge the gaze of angry, fearful, and neutral faces across a range of gaze directions. The result showed: (1) the perception of gaze direction was influenced by facial expression and this was modulated by trait anxiety. For the high trait anxiety group, the CoDG for angry expressions was wider than for fearful and neutral expressions, and no significant difference emerged between fearful and neutral expressions; For the low trait anxiety group, the CoDG for both angry and fearful expressions was wider than for neutral, and no significant difference emerged between angry and fearful expressions. (2) Trait anxiety modulated the perception of gaze direction only in the fearful condition, such that the fearful CoDG for the high trait anxiety group was narrower than the low trait anxiety group. This demonstrated that anxiety distinctly affected gaze perception in expressions that convey threat (angry, fearful), such that a high trait anxiety level modulated the impact of indirectly threatening expressions (fearful), and did not influence responses to directly threatening expression (angry). These findings partially support the *shared signal hypothesis*.

## Introduction

The human face portrays vast quantities of information to facilitate interpersonal communication. Among the various sources of information, eye gaze direction plays a major role in face processing (Framorando et al., 2017) and social communication. By observing other’s eye gaze direction we can speculate what a person is looking at and where her/his attention is focused. Judging other’s eye gaze direction is thus an important skill for human’s survival and social communication. The judgment of other’s eye gaze direction is not highly accurate, however. People have a bias to judge other’s eye gaze as looking at them, even if it is looking away (Stoyanova et al., 2010; Mareschal et al., 2013). The propensity to judge others’ eye gaze direction as directed at the self is influenced by facial expression, especially facial expressions related to threat, such as anger and fear portrayals. Recent studies demonstrate that an angry facial expression was more likely to be judged as directly gazing at the perceiver, whereas a fearful facial expression was more likely to be judged as averting its gaze from the perceiver (Lobmaier et al., 2008; Slepian et al., 2011). As this research has progressed, an index was produced, commonly known as the cone of direct gaze (CoDG) (Gamer and Hecht, 2007), which characterizes the range of eye deviations that participants judge as being directed toward themselves. A wider CoDG indicates that participants were more prone to judge other’s eye gaze as directed at themselves. Ewbank et al. (2009) found that the CoDG of anger was wider than that of fear (Rhodes et al., 2012; Jun et al., 2013). The results showed individuals were prone to judge angry face looking at themselves, and fearful face looking away.

One explanation for this divergent impacts of anger and fear on gaze direction judgment is the *shared signal hypothesis*. According to the hypothesis, both emotion and eye gaze behavior are associated with behavioral motivational orientations to approach or avoid (Adams and Kleck, 2003). Angry and direct gaze are approach oriented, whereas fear and averted gaze are avoidance oriented. When judging eye gaze direction, individuals may be more likely to judge the eye direction in a manner congruent with the signal value of the facial expression. Thus the gaze direction of an angry face is more likely to be judged as direct, and the gaze direction of a fearful face is more likely to be judged as averted.

Although supported by some evidence (Milders et al., 2011; Artuso et al., 2012; Ricciardelli et al., 2012; Rigato et al., 2013), the *shared signal hypothesis* was questioned by numerous studies examining judgments of fearful expressions. Previous studies found the processing of a fearful face was not enhanced under an averted gaze condition (Willis et al., 2011), but was enhanced under direct gaze condition (Bindemann et al., 2008; Slessor et al., 2010). Also, some research found the gaze direction of fearful face is not more likely to be judged as averted compared to the judged gaze direction of an angry face (Lobmaier and Perrett, 2011; Rimmele and Lobmaier, 2012; Schulze et al., 2013).

As stated above, angry facial configurations have a consistent influence on the processing of direct gaze, whereas divergent evidence exists for the impact of fearful expressions on the processing of averted gaze. A possible reason for these inconsistent results is that these studies didn’t control for perceiver characteristics—namely, the anxiety level of the participants. Recent studies demonstrate that the interaction of facial expression and gaze direction differs in high- versus low-anxiety individuals. On the one hand, high and low anxiety level individuals have different responses to fearful faces. High anxiety individuals demonstrate a gaze cuing effect: they deployed attention toward a target located by fearful faces more quickly than by neutral faces. In contrast, this gaze cueing effect was not present in low anxiety individuals (Mathews et al., 2003; Holmes et al., 2006; Fox et al., 2007). Further, the anxiety level of participants was positively related to the strength of gaze cuing effect induced by fearful faces (Putman et al., 2006). On the other hand, individual anxiety levels could modulate the perception of others’ eye gaze direction more globally. A recent study showed a positive, linear relationship between self-reported social anxiety and stronger self-directed perception of others’ gaze directions (Schulze et al., 2013). And the CoDG for socially anxious individuals was wider than in normal controls when a second person was present (Gamer et al., 2011; Harbort et al., 2013).

Divergent effects across the literature might be accounted for by differing anxiety levels in the participants sampled. An inconsistent effect of fearful facial actions on gaze perception would be observed across samples if the average level of anxiety differed across samples. Here, we explore the effect of an individual’s trait anxiety level on the interaction between eye gaze perception and facial expression to help to resolve this discrepancy in the previous literature.

As far as we know, three prior studies have tested the modulation of anxiety on the interaction between facial expression and eye gaze direction. Jun et al. (2013) investigated the effect of expression (anger, fear, and neutral) on the CoDG in social anxiety. They found angry and neutral faces elicited wider cones than fearful faces, but didn’t observe an interaction effect between anxiety and expression, such that expression modulated the CoDG consistently across low- and high-anxiety groups. Schulze et al. (2013) used a web-based approach to explore whether facial expressions (angry, fearful, happy, and neutral) modulated perception of gaze directions in social anxiety. They found a positive, linear relationship between self-reported social anxiety and stronger self-directed perceptions of others’ gaze for both negative and neutral expressions. Critically, this research also didn’t reveal a distinct influence of anxiety on the perception of eye-gaze direction for different negative expressions (angry, fearful). Harbort et al. (2013) similarly found that the individual’s level of anxiety modulated the effect of facial expression (anger, happy and neutral) on the CoDG (in Experiment 2). However, they used only one type of negative expression (anger), and didn’t explore the modulation of anxiety on the CoDG of fear.

In addition, previous studies did not measure (Jun et al., 2013; Schulze et al., 2013) or match depression scores across individuals with different levels of anxiety (Harbort et al., 2013). Anxiety is often accompanied by depression. However, anxiety and depression are associated with different cognitive patterns (Heller et al., 1995; Mogg and Bradley, 1998; Dibbets et al., 2015). Anxious individuals have a cognitive bias toward threatening stimuli, but depression in anxious individuals has been shown to attenuate this bias (Mathews and Mackintosh, 1998). So depression might be a critical factor that dampens the modulatory effect of anxiety on eye gaze direction perception in facial expressions. We suggest that the lack of control for depression levels in the prior literature may account for inconsistent anxiety effects.

The current study attempted to reexamine the effect of trait anxiety on the processing of gaze direction perceived in facial expressions by matching on depression scores. We used the same experimental task as Ewbank et al. (2009). Participants were stratified into high/low trait anxiety groups and asked to judge the gaze of angry, fearful, and neutral faces across a range of gaze directions (-9, -6, -3, 0, 3, 6, 9 pixels). A reliable psychophysical index of the range of eye gaze angles judged as being directed at oneself (CoDG) was used as the dependent variable in this study.

We hypothesize that the modulatory effect of anxiety on perceptions of gaze direction across distinct negative expressions will be observed when anxiety is investigated independent of depression. To verify this hypothesis, the present study ruled out the influence of a depression difference between high and low trait anxiety participant by matching the two groups on depression scores. After controlling the depression score of high and low trait anxiety participants at a low-level, we expected to observe that trait anxiety modulated gaze direction perception depending on the expression type. We further predicted that the CoDG of fearful expressions would be modulated by perceiver trait anxiety, whereas the CoDG of anger expression would not be modulated by perceiver trait anxiety. This hypothesis builds on convergent evidence showing stable patterns in gaze direction perception for anger expressions but variable patterns of gaze direction perception for fearful expressions depending on different experimental paradigms and tasks.

## Materials and Methods

### Participants

Before conducting the experimental task, 1218 individuals completed the Chinese version of ZUNG Anxiety self-assessment scale. 180 participants, whose ZUNG anxiety scores were in the highest 10% and lowest 10%, were invited to participate in lab testing. Before lab testing, all participants completed the trait version of the State-Trait Anxiety Inventory (STAI; Spielberger, 1983) and the Chinese version of the Beck Depression Inventory (BDI). According to their STAI anxiety scores, participants who scored 38 or below were categorized into the low trait anxiety group, and 39 or more into the high trait anxiety group. One hundred and six volunteers (see **Table 1**) were matched on their depression scores across the low and high trait anxiety groups and were invited to participate in the formal experiment. All participants reported normal or corrected-to-normal vision. Two participants were eliminated because of failure in data fitting. Altogether, the data of 52 low trait anxiety participants (25 female, 27 male; *M* = 20.08 years, *SD* = 1.12) and 52 high trait anxiety participants (20 females, 32 males; *M* = 20.04 years, *SD* = 1.12) were processed in the final statistical analysis. After the experiment, they were paid for participating. The study was approved by the ethics committee of Liaoning Normal University. Signed informed consent was provided by each participant prior to participation, and all procedures were in compliance with the Code of Ethics of the World Medical Association (Declaration of Helsinki).

**Table 1:** Participant descriptives on trait anxiety and depression measures.

	Low trait anxiety group (*n* = 53)	High trait anxiety group (*n* = 53)	*p*
ZUNG Anxiety	37.12 (6.56)	43.50 (9.78)	0.000
STAI-Trait	33.77 (2.75)	44.65 (4.02)	0.000
BDI	6.96 (3.77)	7.73 (4.37)	0.242


Trait anxiety and depression scores for low trait anxiety group and high trait anxiety group (Means and Standard Deviations in parentheses).

### Apparatus

The study was run on personal computers running on Windows XP using E-prime 1.0 software. The stimuli were presented on a screen with a resolution of 1024^∗^768 pixels and a color depth of 32 bits.

### Stimuli

Images were grayscale photographs of seven Chinese males. Each of the seven male faces portrayed four emotions: neutral, happy, anger, fear, a total of 28 face images. Non-facial areas and hair were masked, leaving only the central face area visible. All facial images were reduced in size: 213 pixels in width and 314 pixels in height, and subtended a visual angle of 8.14° by 9.74° on screen.

In order to ensure all the facial images conform to standardization requirements (i.e., as tokens of the emotion portrayed) before the formal experiment, 18 female volunteers were recruited to evaluate three aspects of the facial images: emotional category, arousal, and valence.

#### Emotional Category Evaluation

Each trial was initiated by a central fixation cross presented for 500 ms. Then a face was presented which remained until the participant’s response. In the bottom of the screen, there were five response options: neutral, happy, angry, fearful, surprise. Participants were asked to judge “which expression does the face show?,” and answered by clicking the corresponding option on the screen with the mouse cursor. The next trial was initiated automatically following the participant response. All images were presented twice in random order.

#### Arousal Evaluation

The procedure was same as the *emotional category evaluation*, with the exception of the judgment type. Participants were asked “when you see the picture, how do you feel, sleepy, or highly excited?” Rating was made on nine-point continuous scales ranging from 1 (sleepy) to 9 (highly excited). There were nine number options under the face, and participants answered by clicking the corresponding number option on the screen with the mouse cursor.

#### Valence Evaluation

The procedure is the same as the *emotional category evaluation* and the *arousal evaluation*, with the difference that participants were asked “when you see the picture, how do you feel, unpleasant or pleasant?” They were required to choose a number from 1 (unpleasant) to 9 (pleasant) to represent their experience of the picture.

Based on the results of the *emotional category evaluation* pilot, the best tokens (i.e., judged consistently with the category portrayed) were selected respectively for the three types of emotional images (neutral, angry, fearful) (see **Table 2**). This yielded a set of facial images. The results of *valence evaluation* and *arousal evaluation* indicated that the anger facial tokens and fear facial tokens did not significantly differ in valence or arousal level (valence: *t*_(34)_ = 0.286, *p* = 0.776; arousal: *t*_(34)_ = -1.06, *p* = 0.297).

**Table 2:** Results of Pilot Stimuli Evaluation Tasks.

	Neutral	Angry	Fearful
*Emotional category evaluation (%)*	0.98 (0.08)	0.88 (0.17)	0.82 (0.20)
*Valence evaluation*	5.12 (0.28)	2.81 (1.03)	2.71 (1.10)
*Arousal evaluation*	3.50 (1.17)	5.22 (1.75)	5.92 (2.16)


The *Emotional category evaluation*, *Valence evaluation*, and *Arousal evaluation* average ratings from 18 female raters of the three images that were selected for the main experiment for each emotion (Means and Standard Deviations in parentheses).

**FIGURE 1:**
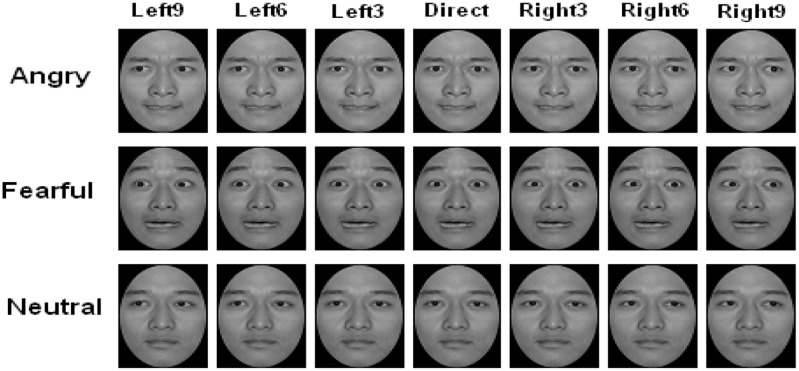
Example of the stimulus faces for three expressions (angry, fearful, and neutral) in seven different gaze directions: 9 pixels to the left, 6 pixels to the left, 3 pixels to the left, direct gaze, 3 pixels to the right, 6 pixels to the right, 9 pixels to the right. The owner of example facial images consented to publish his portrait in any academic journals.

We manipulated gaze by altering the position of the iris of each eye using Adobe Photoshop. We used a total of seven gaze deviations: true direct gaze plus shifts of 3, 6, and 9 pixels for left and right gaze (see **Figure 1**). A total of 63 facial images were used in the formal experiment.

### Task and Procedure

A 2 × 3 × 7 mixed design was used, where the between-subjects factor was Anxiety Group (high trait anxiety group, low trait anxiety group), and the within-subjects factors were the emotion portrayed by a face (neutral, angry, fearful) and gaze direction (-9, -6, -3, 0, 3, 6, 9). Participants were seated 60 cm in front of a computer monitor. A chin rest was used to maintain head position and distance from the screen. Each trial began with a central fixation cross, which remained on-screen for 1000 ms. The fixation cross was followed by a centrally presented face for 500 ms. The face was displayed on a black background. Following the offset of the face, there were three buttons: looking to my left, looking at me, and looking to my right. The buttons remained on screen until the participant rendered a response. Participants were required to click one of three buttons. Participants were instructed to concentrate on categorizing the direction of the gaze as accurately as possible; speed of response was not emphasized. All stimuli were shown five times, in random order, and no feedback was given. The experiment contained 315 trials and lasted approximately 20 min. Prior to the start of the experiment, all participants were given a 14-trial training block. The training facial images were from a neutral female face with three gaze directions (left, direct, right).

### Measuring CoDG

The CoDG were measured using a similar method to Ewbank et al. (2009). We used Matlab to fit logistic functions for the proportion of left, right, and direct responses by the emotion conditions for each participants, separately. A function for “direct” responses was calculated by subtracting the sum of “left” and “right” responses from one. The CoDG was taken as the distance (in pixels of gaze deviation) between the points that two averted curves intersected with the “direct” curve: the left-direct response intersection and the right-direct response intersection.

## Results

A two-way repeated measures analysis of variance (ANOVA)was calculated on CoDG with anxiety group (high trait anxiety group, low trait anxiety group) as the between-participants factor and emotion portrayed (neutral, angry, fearful) as the within-participants factor. This analysis revealed a main effect of emotion, *F*(2,204) = 10.772, *p* < 0.001, $\eta_{p}^{2}$ = 0.096, and a interaction effect between emotion and group, *F*(2,204) = 3.469, *p* < 0.05, $\eta_{p}^{2}$ = 0.033. No significant main effect of group were found in the ANOVA, *F*(1,102) = 1.149, *p* > 0.05.

Pair-wise comparisons (Bonferroni-corrected) across the three facial expression conditions revealed a wider CoDG for angry (8.12 ± 2.04, M ± SD) than fearful expressions (7.68 ± 1.94), *p* < 0.05, and neutral expressions (7.46 ± 2.16), *p* < 0.05; There was no significant difference between fearful and neutral expressions, *p* > 0.05.

To compare CoDG between the high trait anxiety group and the low trait anxiety group in each emotion condition, independent samples *t*-tests were conducted on CoDG in the three emotion conditions. In the fearful condition, a wider CoDG was observed for the low trait anxiety group than for the high trait anxiety group, *t*_(102)_ = 2.215, *p* < 0.05, Cohen’s *d* = 0.43. There was no significant difference between the high trait anxiety group and low trait anxiety group in the angry and neutral conditions (angry: *t*_(102)_ = 0.482, *p* > 0.05; neutral: *t*_(102)_ = 0.353, *p* > 0.05).

**FIGURE 2:**
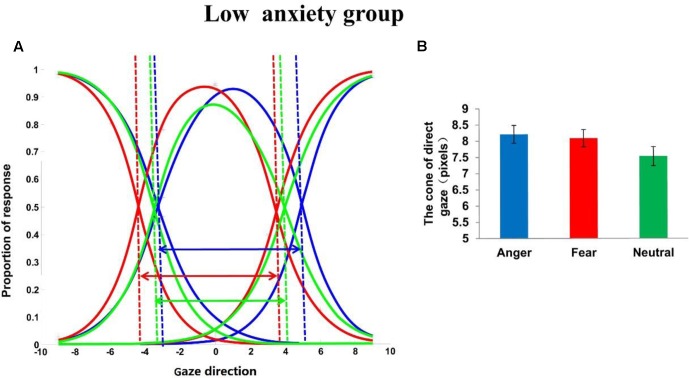
**(A)** Plot showing mean fitted logistic functions for left, direct, and right responses for anger (blue lines), fear (red lines), and neutral (green lines) expression conditions in the low trait anxiety group. Dashed lines show cross-over points used to calculated cone of gaze. Arrows represent width of cone. **(B)** Mean width of cone for anger, fear, and neutral expressions in the low trait anxiety group. Error bars represent standard errors of the mean.

**FIGURE 3:**
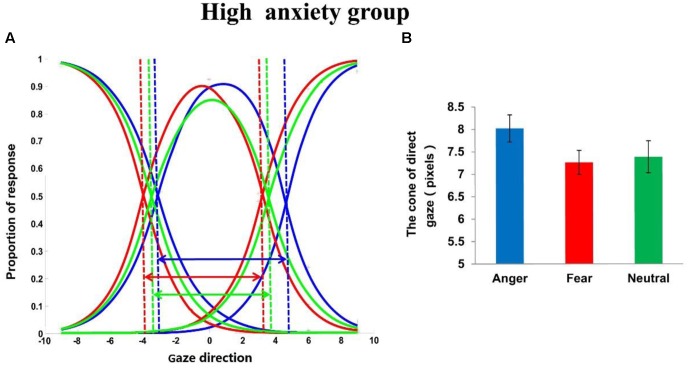
**(A)** Plot showing mean fitted logistic functions for left, direct and right responses for anger (blue lines), fear (red lines), and neutral (green lines) expression conditions in the high trait anxiety group. Dashed lines show cross-over points used to calculated cone of gaze. Arrows represent width of cone. **(B)** Mean width of cone for anger, fear, and neutral expressions in the high trait anxiety group. Error bars represent standard errors of the mean.

To explore the effect of emotion (angry, fearful, neutral) in the high trait anxiety group and the low trait anxiety group separately, two one-way repeated measures ANOVAs on CoDG, with emotion as the within-participants factor, were also calculated separately. For the low trait anxiety group (see **Figure 2**), this analysis revealed a main effect of emotion, *F*(2,102) = 5.953, *p* < 0.01, $\eta_{p}^{2}$ = 0.105. Pair-wise comparisons (Bonferroni-corrected) across the emotions revealed that the CoDG was wider for angry expressions (8.21 ± 2.02)than for neutral expressions, *p* < 0.05. The CoDG for fearful expressions (8.09 ± 1.91)was also wider than that for neutral expressions (7.54 ± 2.16), *p* < 0.05; No significant difference between the CoDG for angry and fearful expressions was observed, *p* > 0.05. For the high trait anxiety group (see **Figure 3**), this analysis revealed a main effect of emotion, *F*(2,102) = 8.427, *p* < 0.001, $\eta_{p}^{2}$ = 0.142. Pair-wise comparisons (Bonferroni-corrected) across the emotions revealed that the CoDG for angry expressions (8.02 ± 2.07) was wider than for fearful (7.26 ± 1.91), *p* < 0.05, and neutral expressions (7.39 ± 2.18), *p* < 0.05. No significant difference between the CoDG for fearful and neutral expressions was observed, *p* > 0.05.

## Discussion

The current study investigated whether trait anxiety level modulates the effect of facial expression on the perception of eye-gaze direction. We used the same experimental task as Ewbank et al. (2009). Participants were stratified into high and low trait anxiety groups and asked to judge the gaze direction of angry, fearful and neutral faces for which the gaze direction was varied. The results revealed that trait anxiety modulated the effect of facial expression on the perception of gaze direction. For the low trait anxiety group, the CoDG for angry and fearful faces was wider than for neutral faces, and no significant difference between angry and fearful faces was found; For the high trait anxiety group, the CoDG for angry faces was wider than for fearful and neutral faces, with no significant difference between fearful and neutral. By matching across our low and high trait anxiety groups by depression level, the present study demonstrated that our findings are related to the trait anxiety of individuals rather than confounded differences in depression level.

Our study demonstrates that emotional expressions affect the perception of eye-gaze direction (for individuals with low and high trait anxiety, although in different patterns) demonstrating that the processing of facial expression and eye gaze direction are interdependent. This finding is consistent with previous work (Ganel et al., 2005; Graham and LaBar, 2007; Lobmaier et al., 2008; Ewbank et al., 2009; Slepian et al., 2011; Rhodes et al., 2012; Harbort et al., 2013; Jun et al., 2013), in which participants were unable to ignore expression when classifying the gaze or ignore gaze when classifying the expression (Ganel et al., 2005; Graham and LaBar, 2007). The perception of eye-gaze direction is influenced by emotional expression (Lobmaier et al., 2008; Ewbank et al., 2009; Slepian et al., 2011; Rhodes et al., 2012; Harbort et al., 2013; Jun et al., 2013), and the perception of emotional expression is influenced by eye-gaze direction (Adams and Kleck, 2003, 2005; Sander et al., 2007; Milders et al., 2011; Ricciardelli et al., 2012). In addition, a wider CoDG for angry faces than neutral demonstrates a bias when processing negative facial expressions, especially angry expressions, which may convey a direct threat. Detecting a direct threat has important survival significance – the bias to judge angry expressions as self-directed may reflect an effort to maximize correct detections of threat. For direct threat information, a miss (not detecting a threat when it is present) may be far more costly than a false alarm (detecting a threat when it is not present).

The present study also revealed that trait anxiety modulated perceptions of gaze direction differently depending on the facial expression (angry or fearful). The CoDG for fearful faces was narrower than for angry faces in the high trait anxiety group, but no difference between the CoDG of fearful and angry faces was observed in the low trait anxiety group. While angry and fearful facial expressions may convey information about threat, the orientation of the threat is different. An angry face can convey a direct threat—that is, the threat may originate from the individual making the facial expression. A fearful face conveys an indirect threat, indicating that the threat is originating from the surrounding environment. In the present study, high trait anxiety individuals more likely judged the gaze direction of angry faces as looking at themselves, and more likely judged the gaze direction of fearful faces as looking away; low trait anxiety individuals are prone to judging the direction of gaze as looking at themselves in both emotional conditions (anger and fear). This finding reveals that high trait anxiety individuals are prone to judge the direction of gaze as consistent with the potential source of a threat based on the specific expression type (the threat of the angry face is derived from the face, the threat of fearful face is derived from the surrounding environment). Low trait anxiety individuals were more likely to judge the direction of gaze consistently with the direction of threat source only when the threatening information was obvious (angry faces); when the threatening information was not obvious (fearful faces), they did not judge the direction of the gaze consistently with the threat source.

Finally, we observed that the CoDG for the high trait anxiety group was narrower than that for low trait anxiety group only for fear expressions (no difference was observed for anger). This finding demonstrates that high trait anxiety individuals were likely to judge the gaze direction of a fearful face as oriented toward the surroundings compared with low trait anxiety individuals. There are two potential explanations for this finding: first, as outlined above, a fearful face may imply a threat that comes from the surrounding environment. Highly trait anxious individuals may allocate more attention resources to detecting threats (Holmes et al., 2006; Fox et al., 2007), and this is more clearly signaled by a fearful face oriented outward. Thus, it would be more likely for highly trait anxious individuals to monitor for (and potentially missperceive) fearful faces as looking away instead of looking at themselves, producing a narrower CoDG. A second explanation is that highly trait anxious individuals do not think they are the reason for the fear of others, perhaps because they do not experience themselves as agentic/powerful (i.e., their appraisals of the situation differ from individuals with low anxiety). Individuals who are highly trait anxious may rarely experience other people’s fear directed at themselves. As a result, they may tend to use this prior experience to assume that another individual is afraid of something else in the environment.

Our findings partially support the *shared signal hypothesis* (Adams and Kleck, 2003, 2005), which proposes that gaze behavior and emotion are associated with the behavioral motivation to approach or avoid. According to this view, anger and direct gaze are associated with approach motivation while fear and averted gaze are associated with avoidance motivation. The *shared signal hypothesis* predicts that the congruent pairs (anger face paired with direct gaze, or fearful face paired with averted gaze) would be processed more efficiently than incongruent pairs (anger face paired averted gaze, or fearful face paired direct gaze). In this case, individuals should tend to judge the angry face looking at themselves and judge the fearful face looking away, and the neutral face is in the middle of the two expressions. However, the current results demonstrate a more nuanced pattern such that the CoDG of anger expressions was wider than that of fear expressions, only in the high trait anxiety group. Furthermore, inconsistent with the *shared signal hypothesis* the CoDG of neutral expressions was not in the middle of anger and fear expressions (in both high and low trait anxiety group conditions).

## Conclusion

The current study matched the depression scores across two groups of high and low trait anxious participants to investigate the effect of trait anxiety on the CoDG of different threatening expressions. The CoDG for anger expressions was wider than for neutral expressions; but the pattern of effects on the CoDG of different threatening expressions depends on the trait anxiety level of participants. Trait anxiety enhanced the bias for indirectly threatening information (fear expressions were judged to have a narrower CoDG), but not for directly threatening information (the CoDG did not differ for anger expressions). Our findings partially support the *shared signal hypothesis.*

## Author Contributions

ZH was responsible for the design of experiment, data analyze and paper writing. MG was responsible for modifying the language problem of the article. QL was responsible for writing the experimental program and analysis method. GZ was responsible for data collection. HL was responsible for the design of experiment.

## Conflict of Interest Statement

The authors declare that the research was conducted in the absence of any commercial or financial relationships that could be construed as a potential conflict of interest.
